# Integration of Magnetocardiography and Coronary Computed Tomography Angiography With Machine Learning for Detection of Functionally Significant Myocardial Ischemia

**DOI:** 10.31083/RCM47473

**Published:** 2026-06-08

**Authors:** Xincheng Li, Shuangxiang Lin, Linlin Sun, Yanli Yu, Haipeng Liu, Zhen Wang

**Affiliations:** ^1^The Fourth School of Clinical Medicine, Zhejiang Chinese Medical University, Hangzhou First People's Hospital, 310000 Hangzhou, Zhejiang, China; ^2^Department of Radiology, The Second Affiliated Hospital, Zhejiang University School of Medicine, Zhejiang Chinese Medical University, 310000 Hangzhou, Zhejiang, China; ^3^Department of Radiology, Affiliated Hangzhou First People's Hospital, Westlake University School of Medicine, 310000 Hangzhou, Zhejiang, China; ^4^Centre for Intelligent Healthcare, Coventry University, CV1 5RW Coventry, UK

**Keywords:** coronary artery disease, myocardial ischemia, magnetocardiography, computed tomography angiography, machine learning

## Abstract

**Background:**

Functional assessment of myocardial ischemia is essential and can be evaluated noninvasively using coronary computed tomography angiography (CCTA) and magnetocardiography (MCG). However, the diagnostic value of integrating CCTA and MCG has not been investigated.

**Methods:**

This retrospective, single-center cohort study included 275 patients with suspected coronary artery disease (CAD) who underwent both CCTA and MCG examinations from December 2023 to June 2025. Functionally significant ischemia was defined by invasive fractional flow reserve (FFR) or CT-derived FFR (CT-FFR). Quantitative features from both modalities were extracted and normalized. Machine learning (ML) models based on MCG alone, CCTA alone, and combined MCG–CCTA were constructed and evaluated using five-fold cross-validation. Model performance was assessed using the area under the receiver operating characteristic curve (AUC), accuracy, sensitivity, and specificity; model interpretability was examined using Shapley additive explanations (SHAP).

**Results:**

Of the 275 patients, 98 (35.6%) were classified as being ischemic. The MCG model achieved an AUC of 0.769 (95% confidence interval (CI): 0.708–0.829), and the CCTA model yielded an AUC of 0.755 (95% CI: 0.692–0.818). In contrast, the combined MCG–CCTA model developed using ML demonstrated superior performance, with an AUC of 0.829 (95% CI: 0.773–0.885), an accuracy of 0.800, a sensitivity of 0.704, and a specificity of 0.853.

**Conclusions:**

A combined MCG–CCTA model developed with ML outperforms models based on either modality alone for detecting functionally significant myocardial ischemia. In clinical practice, this integrated approach may enhance ischemia assessment and inform downstream testing decisions.

## 1. Introduction

Coronary artery disease (CAD) stands as a major global health challenge and the foremost cause of mortality worldwide [1]. Myocardial ischemia, which induces regional wall motion abnormalities and diminishes cardiac function, significantly worsens the prognosis for CAD [2]. Therefore, precise ischemia assessment is vital for diagnosing stable CAD and determining suitable revascularization strategies [3].

Invasive coronary angiography (ICA) combined with fractional flow reserve (FFR) is the gold standard for evaluating hemodynamically significant stenosis [4]. Nevertheless, its invasive nature and high cost restrict its widespread application. Noninvasive FFR derived from coronary CT angiography (CT-FFR), utilizing computational fluid dynamics (CFD) or machine learning (ML) algorithms, offers a comprehensive, noninvasive evaluation of coronary anatomy and functional parameters. This approach has been validated against ICA with FFR for detecting lesion-specific ischemia [5, 6, 7, 8]. However, severe coronary calcification can introduce blooming artifacts, distorting lumen stenosis assessment and CT-FFR results, thereby compromising diagnostic specificity and potentially leading to unnecessary ICA referrals, which increase both medical risks and healthcare resource burdens [9]. Magnetocardiography (MCG), a noninvasive technique for detecting the exceedingly weak magnetic fields generated by cardiac electrical activity [10], is free from electrode-skin artifacts that plague electrocardiography (ECG). MCG can sensitively capture current changes induced by ischemic myocardium, offering superior sensitivity and specificity [11, 12]. Early studies utilizing superconducting quantum interference devices (SQUID) highlighted MCG’s potential ischemia detection [13], but its clinical adoption has been hampered by the scarcity of liquid helium and high operational costs. Recent advancements in spin-exchange relaxation-free (SERF) atomic magnetometers enable non-contact MCG measurements with enhanced sensitivity without cryogenic cooling, paving the way for large-scale clinical studies and suggesting MCG’s potential as a valuable noninvasive ischemia detection tool [14, 15, 16].

Both coronary computed tomography angiography (CCTA) and MCG provide valuable noninvasive information for detecting myocardial ischemia. However, MCG lacks standardized diagnostic criteria, limiting its broader clinical use. Recent studies have shown that applying ML to MCG can enhance ischemia detection and localization, as well as characterize disease severity and lesion distribution [17, 18]. Yet, the performance of a combined CCTA-MCG model using ML in CAD patients has not been systematically explored.

This study aims to assess whether integrating MCG and CCTA through ML improves noninvasive ischemia diagnosis in patients suspected of having CAD.

## 2. Materials and Methods

### 2.1 Study Design

This single-center, retrospective cohort study included adult patients suspected of having coronary artery disease (CAD) who underwent both CCTA and MCG examinations within a two-week interval from December 2023 to June 2025. CT-FFR data were available for all patients, and a subset underwent invasive FFR. Functionally significant ischemia was defined as CT-FFR ≤0.80. In cases where both CT-FFR and invasive FFR were available, discrepancies were resolved by FFR. This study was conducted in accordance with the revised 2013 Helsinki Declaration and approved by the Ethics Committee of the Affiliated Hangzhou First People’s Hospital, Westlake University School of Medicine (Ethic Approval Number: IIT-20231214-0298-02). Written informed consent was obtained from all participants prior to enrollment.

### 2.2 Study Population

**Fig. 1.**
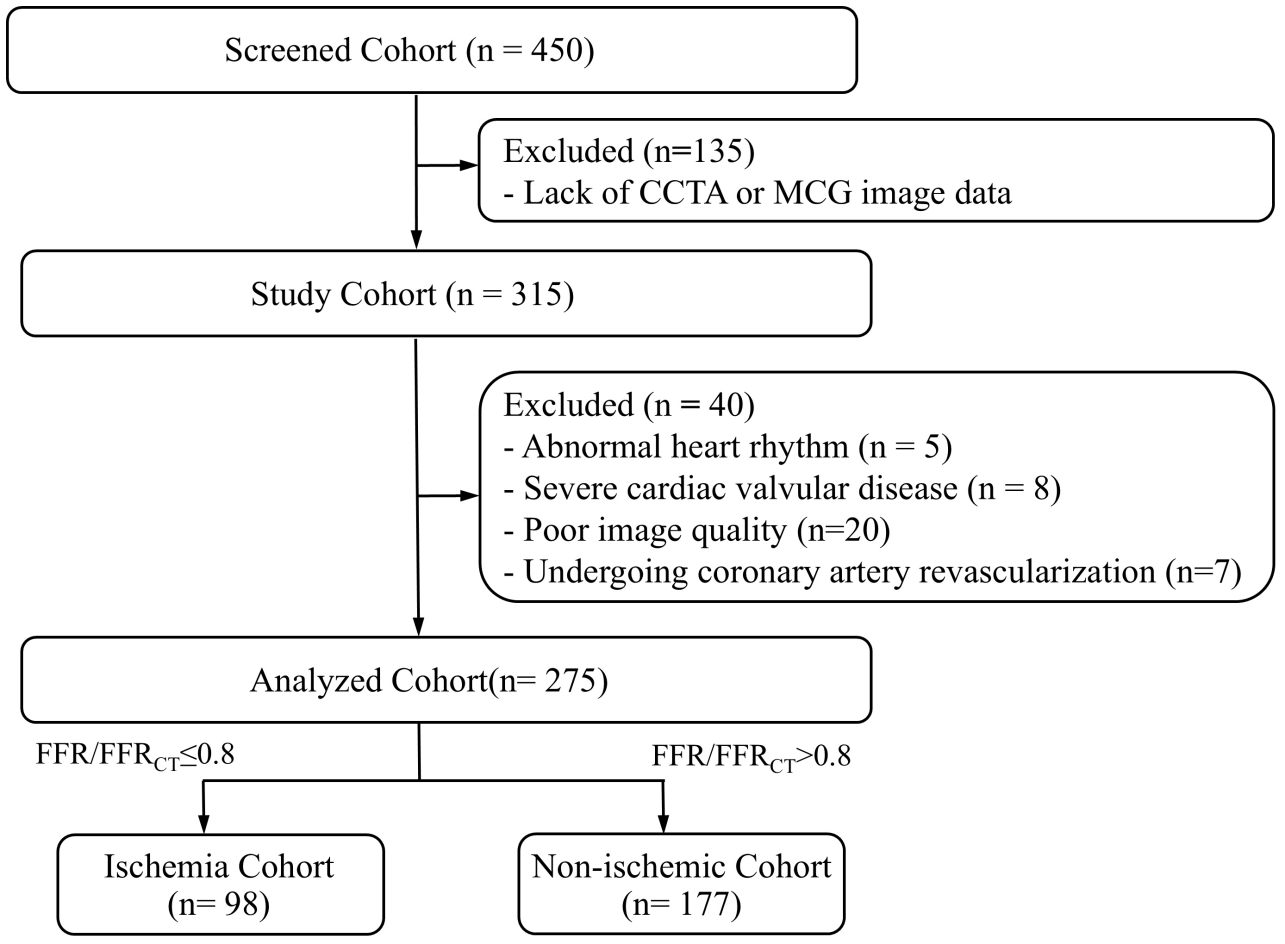
**Flowchart of study population**. CCTA, coronary computed tomography angiography; MCG, magnetocardiography; FFR, fractional flow reserve.

Initially, a total of 450 consecutive patients were screened. The inclusion criteria were as follows: (1) aged over 18 years; (2) presence of angina or angina-like symptoms, such as chest pain or dyspnea, suspected of CAD; (3) completion of both CCTA and MCG within a two-week interval; (4) availability of FFR or CT-FFR measurements. Exclusion criteria included: (1) severe cardiac valvular disease; (2) abnormal heart rhythm; (3) prior coronary artery revascularization; (4) poor image quality; (5) inability to complete MCG acquisition due to intolerance of the magnetic shielding chamber or interference from metallic implants/devices; (6) severe renal dysfunction (estimated glomerular filtration rate (eGFR) <30 mL/min/1.73 m^2^) or dialysis; (7) history of severe hypersensitivity to iodinated contrast media; (8) pregnancy or breastfeeding. After applying these criteria, 275 patients were included in the final analysis. The patient enrollment process is detailed in Fig. 1. All 275 patients had evaluable CT-FFR values, and 12 patients (4.4%) also underwent invasive FFR.

### 2.3 MCG Scan

This study utilized an ultra-high-sensitivity MCG system based on the SERF principle, comprising a 64-channel atomic magnetometer array, a high-performance magnetic shielding chamber, and a multifunctional non-magnetic examination bed. This system can detect cardiac magnetic fields as weak as 10^-7^ times the strength of the Earth’s magnetic field. Data acquisition followed a standardized protocol: subjects were positioned in a supine position on the examination bed, with the sensor array center placed 2 cm below the xiphoid process. Resting MCG signals were continuously recorded for 3 minutes. ECG signals were simultaneously acquired within 5 minutes before and after the MCG scan for heartbeat segmentation and signal comparison. Patients with metallic implants or other strong magnetic interference sources were excluded prior to scanning. During data acquisition, real-time monitoring identified potential issues like body motion, respiratory drift, channel instability, or transient environmental magnetic fluctuations, ensuring only high-quality signal segments were recorded. The system’s performance was regularly validated to ensure stable signal acquisition, with no significant variance observed during repeated scans in the same setting. Additionally, a Signal Quality Score was calculated for all MCG scans, with all patients achieving a score ≥90, ensuring the reliability of the acquired signals. MCG data were collected in a controlled, magnetically shielded environment to minimize external magnetic interference, ensuring high data quality and consistency. This technology offers significant advantages, including non-invasiveness, contactless measurement, and the lack of cryogenic cooling requirements, while enabling high-precision detection of magnetic field variations related to myocardial electrophysiological activity.

### 2.4 CCTA Scan

Coronary CT angiography was performed using a second-generation dual-source CT scanner (SOMATOM Definition Flash, Siemens Healthineers, Forchheim, Germany). Five minutes before scanning, patients received sublingual nitroglycerin (0.5 mg) to dilate the coronary arteries. A non-ionic contrast agent (Iodomane 370 mg/mL, Bayer, Germany, 60 mL) was injected intravenously at 4.5–5.0 mL/s, followed by a 30 mL saline flush at the same rate using a dual-head power injector. Scanning was initiated by automated bolus tracking with a 7-second delay after the attenuation in the ascending aorta reached 100 Hounsfield units. CT scanning was performed during 30%–80% of the R-R interval with prospective ECG gating, and the scanner’s automatic phase selection feature was used to obtain the optimal systolic (33%–46% of the R-R interval) and diastolic (66%–75% of the R-R interval) images. Reconstructions were generated with a slice thickness of 0.75 mm and a field of view of 200–250 mm. Additional acquisition parameters included a tube voltage of 120 kV, a reference tube current of 320 mAs with CARE Dose 4D modulation, a gantry rotation time of 0.28 s per rotation, and a collimation of 64 × 2 × 0.6 mm. This standardized protocol reduced radiation exposure through prospective gating and adaptive phase selection, while dual-phase reconstruction minimized coronary motion artifacts and improved the accuracy of stenosis assessment.

### 2.5 Data Collection and Processing

The multichannel cardiac magnetic signals collected by the MCG system were automatically processed to extract 23 quantitative parameters, including temporal intervals, magnetic field strength indices, as well as angular and spatial dipole metrics (**Supplementary Table 1**). CCTA images were analyzed using an AI-assisted platform (CoronaryDoc, Shukun Technology, Beijing, China), which generated 21 anatomical and functional parameters (**Supplementary Table 2**). For coronary plaque assessment, vessel-level plaque composition was recorded for the left anterior descending artery (LAD), left circumflex artery (LCX), right coronary artery (RCA), and as no plaque, non-calcified, calcified, or mixed plaque. CT-FFR was used solely to define the reference-standard outcome and was not used as an input feature for model development. Parameters with more than 30% missing data were excluded. Missing values, which occurred only in a few continuous variables, were imputed using median substitution. For feature screening, univariate analyses were performed to identify variables potentially related to functionally significant ischemia. Variables demonstrating at least borderline association (*p* < 0.1) were considered candidates for inclusion. Clinically important parameters supported by prior physiological and imaging evidence were also retained to avoid omitting relevant predictors. Feature selection thus followed a combined strategy integrating both statistical signals and clinical relevance, rather than relying solely on *p*-values.

### 2.6 Model Development and Comparison

All 275 patients were included in model development, with five-fold stratified cross-validation used to generate training and validation folds. This framework was consistently applied across three model variants: MCG-only, CCTA-only, and combined MCG-CCTA models. For each model variant, five ML algorithms were implemented: logistic regression (LR), support vector machine (SVM), random forest (RF), naive bayes (NB), and extreme gradient boosting (XGBoost). Model performance was evaluated using the area under the receiver operating characteristic curve (AUC), accuracy, sensitivity, specificity, positive predictive value (PPV), negative predictive value (NPV), and F1 score. Comparative analyses across the three model variants and five algorithms were performed to identify the best-performing configuration for diagnosing functional myocardial ischemia. To enhance interpretability, Shapley additive explanations (SHAP) was conducted to quantify the contribution of individual features to model predictions.

### 2.7 Statistical Analysis

Statistical analyses were performed using SPSS software version 25.0 (SPSS Inc, Chicago, IL, USA) and R software version 4.5.1 (R Foundation for Statistical Computing, Vienna, Austria). The normality of continuous variables was assessed using standard tests for Gaussian distribution. As none of the continuous variables followed a normal distribution, continuous variables are presented as median (interquartile range, IQR), and group comparisons for continuous variables were performed using the Mann–Whitney U test. Categorical variables were compared utilizing the χ^2^ test or Fisher’s exact test, as appropriate. The diagnostic performance of the MCG model, CCTA model, and the combined MCG-CCTA model was evaluated using receiver operating characteristic (ROC) curve analysis, with optimal cutoff values determined by the Youden index. AUC was calculated and compared across models using the DeLong test to evaluate discriminatory ability. Additionally, sensitivity, specificity, PPV, NPV, and accuracy were reported with 95% confidence interval (CI). Two-tailed *p*-values < 0.05 were considered significant.

## 3. Results

### 3.1 Baseline Characteristics

A total of 275 eligible patients were included in this study, comprising 98 (35.6%) patients in the ischemia group and 177 (64.4%) patients in the non-ischemia group. Baseline characteristics are presented in Table 1. The ischemia group had a notably higher proportion of males (78.6% vs. 47.5%, *p* < 0.01), along with a higher prevalence of smoking (26.5% vs. 11.9%, *p* < 0.01), diabetes mellitus (35.7% vs. 18.6%, *p* < 0.01), and hypertension (71.4% vs. 59.3%, *p* = 0.046). In addition, the ischemia group showed lower total cholesterol (4.04 mmol/L vs. 4.41 mmol/L, *p* = 0.012) and high-density lipoprotein cholesterol levels (1.04 mmol/L vs. 1.14 mmol/L, *p* < 0.01). There were no other significant differences between the two groups.

**Table 1.** **Patient baseline characteristics**.

Characteristics		Total cohort (n = 275)	Non-ischemia cohort (n = 177)	Ischemia cohort (n = 98)	*p*-value
Age, years		67.00 (58.00–74.00)	66.00 (56.00–73.50)	68.00 (59.75–75.25)	0.07
Male		161 (58.5%)	84 (47.5%)	77 (78.6%)	<0.01
BMI, kg/m^2^		23.44 (20.76–25.54)	23.31 (20.76–25.55)	23.44 (20.76–25.50)	0.65
History of smoking		47 (17.1%)	21 (11.9%)	26 (26.5%)	<0.01
Hypertension		175 (63.6%)	105 (59.3%)	70 (71.4%)	0.046
Diabetes mellitus		68 (24.7%)	33 (18.6%)	35 (35.7%)	<0.01
Dyslipidemia		25 (9.1%)	15 (8.5%)	10 (10.2%)	0.63
TC, mmol/L		4.26 (3.47–5.02)	4.41 (3.65–5.03)	4.04 (3.15–4.83)	0.012
TG, mmol/L		1.22 (0.90–1.77)	1.18 (0.90–1.70)	1.33 (0.90–1.87)	0.44
HDL-C, mmol/L		1.10 (0.97–1.32)	1.14 (0.99–1.37)	1.04 (0.94–1.23)	<0.01
LDL-C, mmol/L		2.27 (1.58–2.29)	2.35 (1.73–3.01)	2.17 (1.45–2.73)	0.08
PIV, 10^9^/L^2^		467.50 (328.00–669.79)	456.21 (327.20–659.28)	492.75 (332.39–689.54)	0.60
Echocardiography					
	LVIDd, cm	4.73 (4.46–5.00)	4.73 (4.50–5.00)	4.73 (4.44–5.07)	0.90
	LVIDs, cm	2.97 (2.79–3.26)	2.95 (2.79–3.25)	2.98 (2.79–3.27)	0.59
	FS, %	35.70 (33.30–39.40)	35.70 (33.30–39.35)	35.00 (33.18–39.40)	0.68
	LVEF, %	65.10 (60.20–69.90)	64.60 (60.35–69.85)	64.65 (59.33–70.15)	0.73

Data are presented as the median (25–75% interquartile range), and n (%). BMI, body mass index; TC, total cholesterol; TG, triglyceride; HDL-C, high-density lipoprotein cholesterol; LDL-C, low-density lipoprotein cholesterol; PIV, pan-immune-inflammation value; LVIDd, left ventricular internal dimension in diastole; LVIDs, left ventricular internal dimension in systole; FS, fractional shortening; LVEF, left ventricular ejection fraction.

### 3.2 Feature Selection

Univariate analysis revealed that several variables had at least a borderline association (*p* < 0.1) with functionally significant ischemia, including PR interval, ST-segment duration, the R/T magnetic strength ratio, CACS, and plaque composition in the LAD, LCX, and RCA. To ensure no clinically important electrophysiological information was missed, additional MCG parameters relevant to ischemia (QRS interval, QT interval, R-wave peak dipole distance, and T-wave peak dipole distance) were also included. In total, 11 features were used for model development: four CCTA-derived variables (LAD, LCX, RCA plaque characteristics, and CACS) and seven MCG-derived variables (PR interval, QRS interval, QT interval, ST-segment duration, R/T magnetic strength ratio, R-wave peak dipole distance, and T-wave peak dipole distance). The three plaque composition variables were treated as categorical predictors, while CACS and all seven MCG variables were modeled as continuous predictors. These variables were selected not only for their statistical associations but also as recognized indicators of ischemia-related structural and electrophysiological changes, with their mechanistic roles further discussed in the “Discussion” section.

### 3.3 Model Performance and Comparisons

**Fig. 2.**
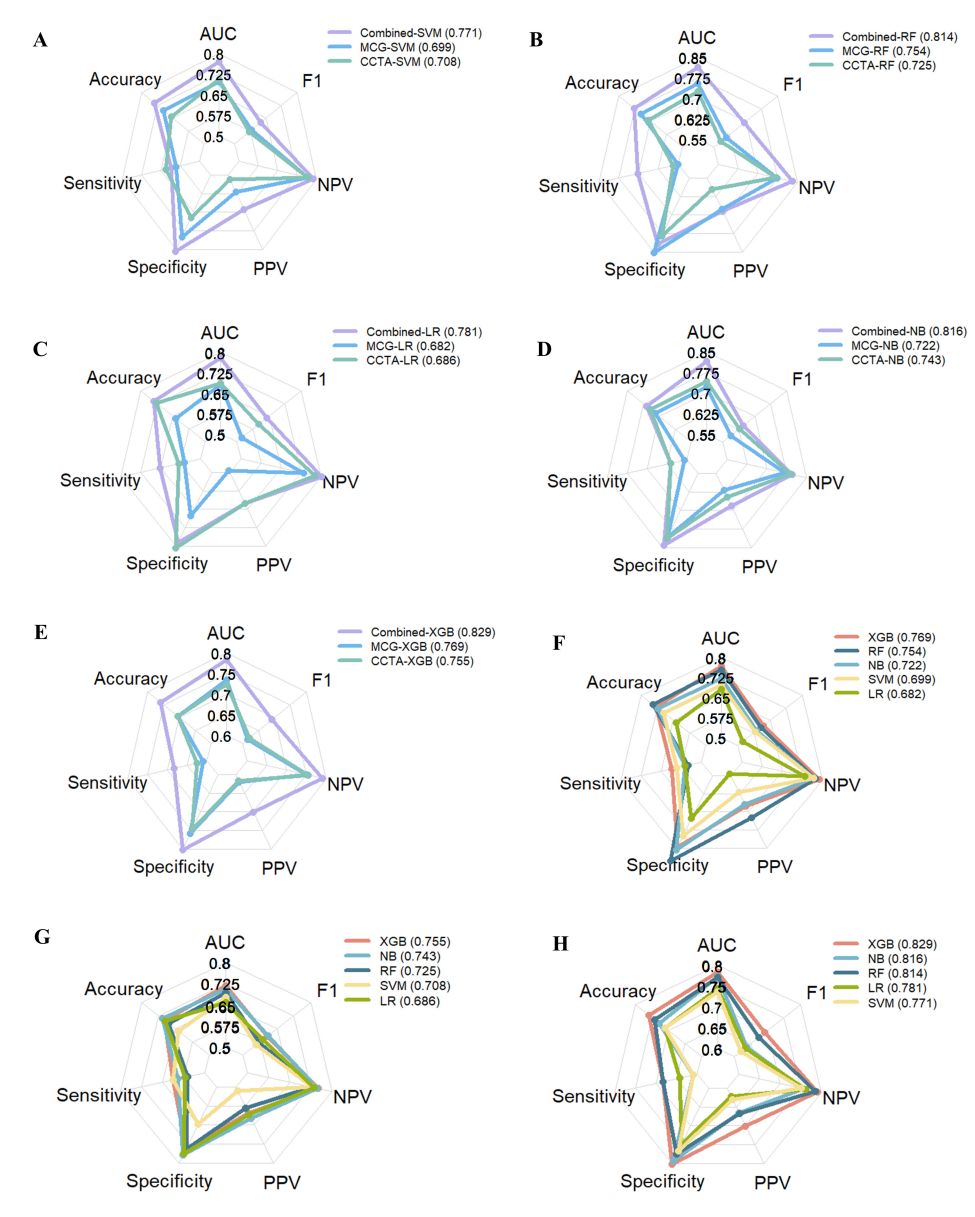
**Radar plots illustrating the diagnostic performance of three models based on five machine learning (ML) methods**. (A) SVM model. (B) RF model. (C) LR model. (D) NB model. (E) XGBoost model. (F) MCG models. (G) CCTA models. (H) Combined models. AUC, area under the receiver operating characteristic curve; F1, F1-score; NPV, negative predictive value; PPV, positive predictive value; SVM, support vector machine; RF, random forest; LR, logistic regression; NB, naive bayes; XGBoost, extreme gradient boosting.

**Fig. 3.**
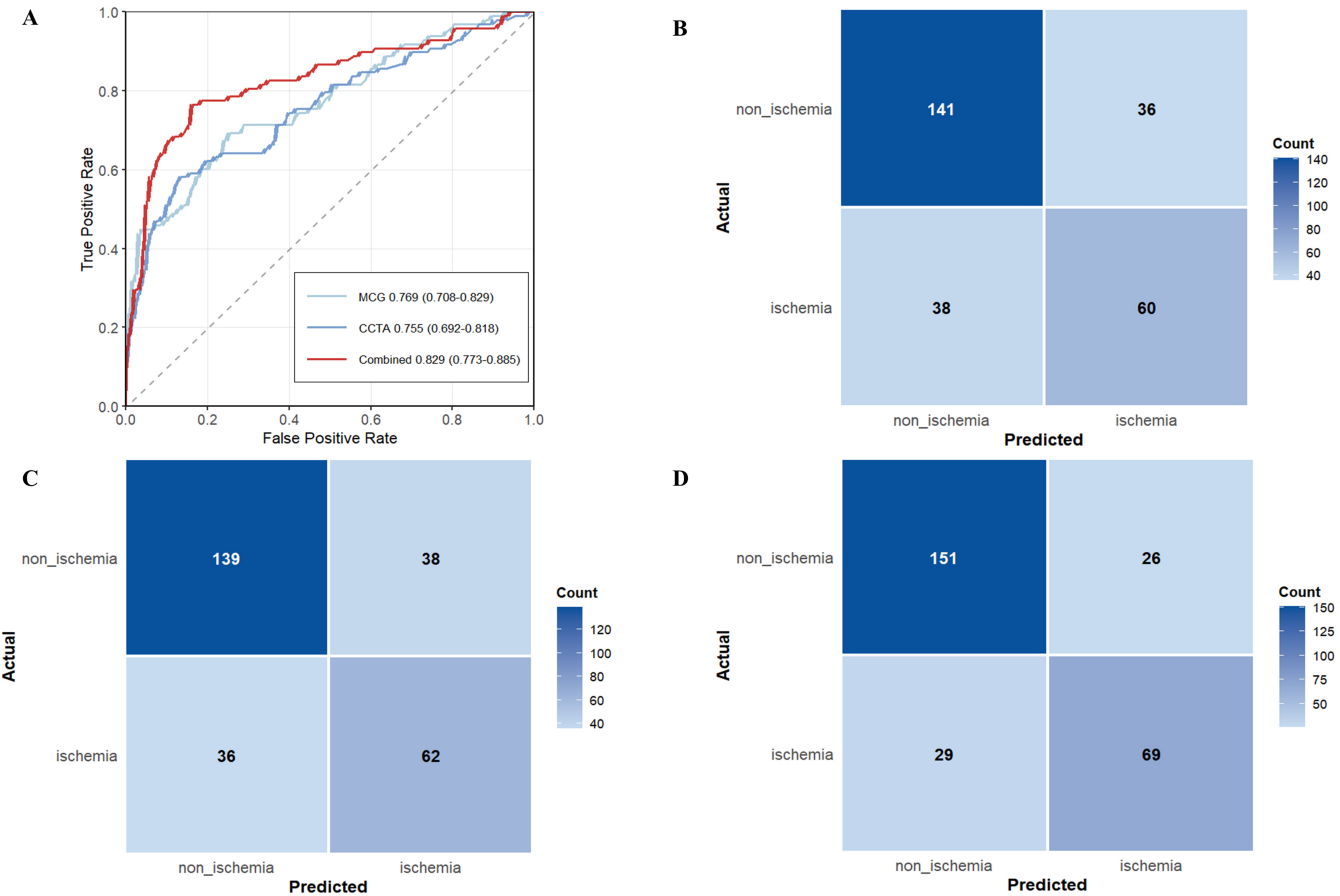
**ROC curves and confusion matrices for MCG, CCTA, and MCG-CCTA models constructed by XGBoost**. (A) ROC curve of the individual MCG model, the individual CCTA model, and the MCG-CCTA fusion model. (B) Confusion matrix analysis for the individual MCG model. (C) Confusion matrix analysis for the individual CCTA model. (D) Confusion matrix analysis for the MCG-CCTA fusion model.

Among the five ML methods tested (LR, RF, NB, SVM, and XGBoost), XGBoost consistently outperformed the others across all modeling strategies (MCG-only, CCTA-only, and combined MCG-CCTA). Overall diagnostic metrics for all algorithms and model types are summarized in **Supplementary Table 3**, and the relative performance profiles are illustrated by radar plots in Fig. 2. For XGBoost models, the MCG-only and CCTA-only configurations achieved AUC values of 0.769 (95% CI: 0.708–0.829) and 0.755 (95% CI: 0.692–0.818), respectively, offering a favorable balance between sensitivity and specificity compared with the other classifiers. Integrating MCG and CCTA further enhanced diagnostic accuracy. The combined XGBoost model delivered the best overall results, with an AUC of 0.829 (95% CI: 0.773–0.885), accuracy of 0.800, sensitivity of 0.704, and specificity of 0.853. This represented relative AUC increases of 7.9% over the MCG model and 9.8% over the CCTA model (DeLong test, all *p* < 0.05). Predictive values were also favorable (PPV = 0.726, NPV = 0.839), resulting in the highest F1-score (0.715). Fig. 3 displays the ROC curves of the MCG-only, CCTA-only, and combined MCG-CCTA XGBoost models, along with their corresponding confusion matrices at the optimal cutoff, which may be helpful as a concise visual summary of discrimination and correct/incorrect classifications. To address the potential impact of confounding by conventional risk factors, we performed subgroup analyses by sex, hypertension, and smoking status. The combined MCG-CCTA model showed similar discrimination across these strata (**Supplementary Table 4**).

### 3.4 Model Interpretation

**Fig. 4.**
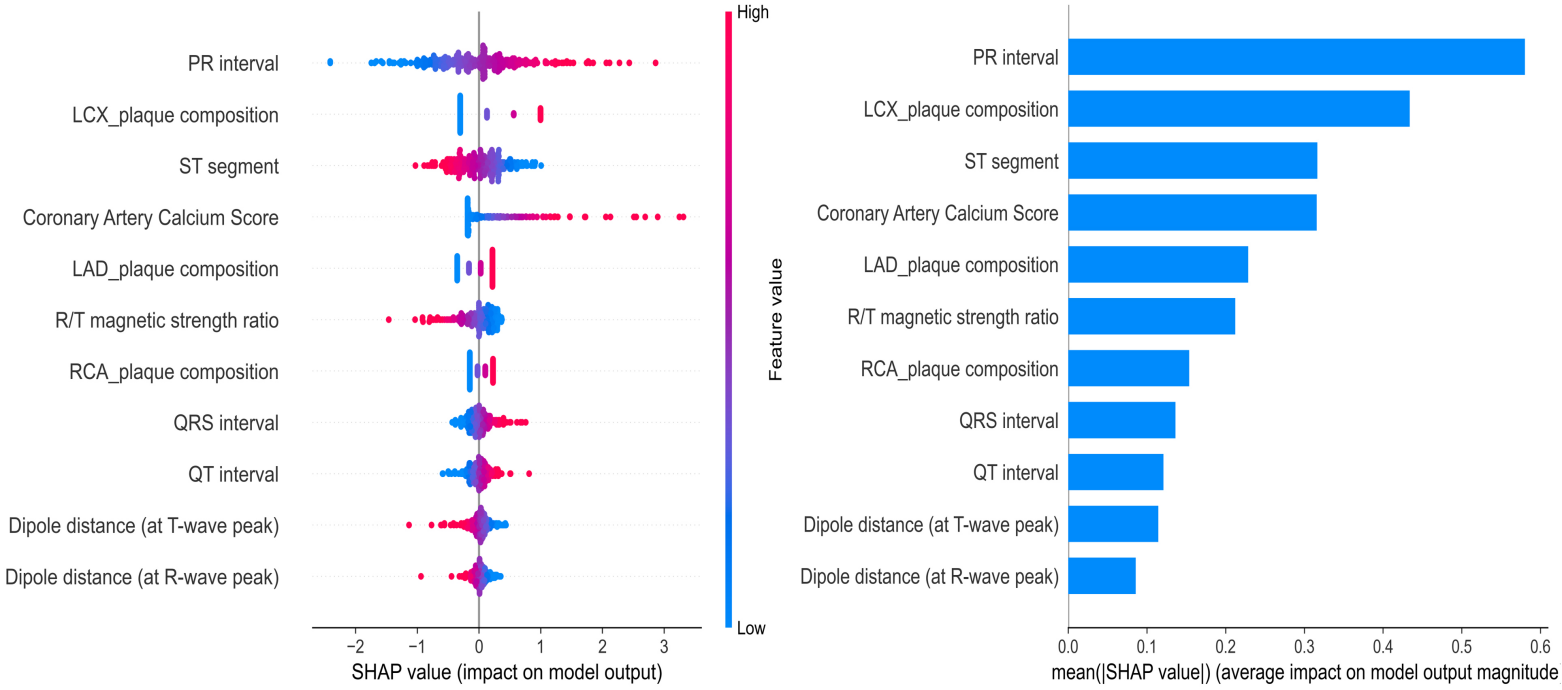
**Global interpretability of the MCG-CCTA combined model constructed by XGBoost using SHAP values**.

SHAP analysis based on the XGBoost combined model (MCG-CCTA fusion) revealed that both MCG and CCTA features significantly contributed to model predictions, confirming their complementary value (Fig. 4). Based on the mean absolute SHAP values, the most influential features were PR interval, LCX plaque composition, ST segment, CACS, and LAD plaque composition, followed by R/T magnetic strength ratio, RCA plaque composition, QRS interval, QT interval, dipole distance at T-wave peak, and dipole distance at R-wave peak. The top five features accounted for approximately 70% of the total feature importance, with MCG-derived timing indices ranking prominently and CCTA-derived plaque composition and calcification measures also making substantial contributions. These findings provide an interpretable explanation for the improved discrimination of the fusion model observed in the performance comparison.

## 4. Discussion

In this study, we developed a machine-learning model that integrates MCG and CCTA to detect functionally significant myocardial ischemia in patients with suspected CAD. By comparing models based on each modality with the combined approach, we found that the XGBoost-based combined MCG-CCTA model achieved the best ischemia discrimination (AUC 0.829), outperforming the MCG model (AUC 0.769) and the CCTA model (AUC 0.755). Our findings suggest that the combined MCG-CCTA model may offer complementary value to current noninvasive ischemia evaluation methods and may help reduce unnecessary invasive testing.

Our findings indicate that MCG or CCTA alone have moderate diagnostic performance for detecting functionally significant ischemia, supporting their individual clinical utility and aligning with prior research. The PLATFORM trial showed that CCTA-based noninvasive functional assessment can streamline diagnostic pathways and reduce unnecessary invasive evaluations [19], while the MAGNETO study demonstrated that MCG can expedite the assessment of patients with suspected ischemia [20].

However, each modality has inherent limitations. Previous studies by Vavere *et al*. [21] and Xu *et al*. [22] reported that severe coronary calcification may lead to artifact-induced overestimation of stenosis severity on CCTA, and He *et al*. [23] suggested that a potential temporal dissociation between hemodynamic disturbances and electrophysiological alterations may cause false-negative MCG results in some patients. In this context, the enhanced performance of the fusion model likely reflects the complementary strengths of the two modalities. Although only a small proportion of patients in our cohort had substantial calcification (CACS ≥400), this subgroup represents a clinically relevant scenario where CCTA interpretation is challenging due to heavy calcification or poor image quality. In such cases, the integrated model can provide complementary information by incorporating electrophysiological ischemia signatures unaffected by calcification-related artifacts. Adding MCG may help refine ischemia assessment and better identify patients who would benefit from or could safely defer invasive angiography. Consistent with this, Wu *et al*. [18] reported that a combined strategy outperformed CCTA alone in heavily calcified patients, though their integration method was primarily additive. Building on these insights, our study incorporated a broader set of electrophysiological and anatomical features through an ML framework, offering a more nuanced synthesis of complementary signals. These findings support the potential role of multimodal fusion in facilitating more informed downstream decision-making and may reduce unnecessary invasive testing, especially when CCTA alone yields indeterminate results.

To further explain the fusion model’s superior discriminative performance, it is important to consider the physiological relevance of the selected features. MCG captures early electrical disturbances caused by myocardial ischemia, including conduction delays and repolarization disturbances [10]. In our feature selection, conduction and repolarization-related temporal indices (PR, QRS, QT) were significantly associated with ischemia, consistent with experimental studies showing that ischemia prolongs atrioventricular and ventricular conduction and alters action potential repolarization duration [24, 25, 26]. Classic electrophysiological ischemia markers such as ST-segment deviation and abnormal R/T magnetic amplitude ratio indicated an imbalance between depolarization and repolarization currents, in line with previous ECG and MCG studies [12]. Spatial dipole features, reflected by the distance between the peak dipoles of the R and T waves, suggested abnormal shifts in activation and recovery vector orientation. Similarly, Han *et al*. [27] reported that dipole displacement corresponded with coronary lesion location and severity. Anatomically, CACS and plaque composition in the LAD, LCX, and RCA formed the structural basis for ischemia, which is consistent with multicenter studies validating the value of CCTA in quantifying plaque burden and assessing calcification [28]. Integrating these electrophysiological and anatomical features provides a coherent mechanistic explanation for the fusion model’s superior diagnostic performance compared to either modality alone.

From a practical standpoint, the present findings should be considered in light of the current availability and cost of MCG. Although MCG is noninvasive, its implementation requires dedicated hardware, a magnetic shielding environment, and trained personnel, which currently may limit its use to specialized centers. The SERF-based platform used in this study eliminates the need for liquid-helium cooling required by conventional SQUID-based systems and may offer advantages in operational simplicity and long-term scalability [16], but infrastructure and workflow requirements remain significant. Therefore, our results support the diagnostic feasibility and potential incremental value of an MCG-CCTA pathway rather than an immediate cost-saving replacement for invasive FFR. Formal health-economic and multicenter validation studies are needed before routine adoption.

## 5. Limitations

There are several limitations in our study. First, it was a single-center retrospective study with a limited sample size, which may restrict the generalizability of the findings. Second, all patients had CT-FFR, with invasive FFR available only in a small subset and used as the reference when present, which may introduce bias. Third, the interval between CCTA and MCG could extend to two weeks, potentially introducing temporal bias owing to changes in ischemic status. Fourth, the small cohort with severe calcification and the lack of detailed plaque features may have limited the depth of related analyses. Fifth, MCG data were obtained from a single SERF-based system at one center, and the lack of inter-scanner evaluation and standardized protocols may affect generalizability.

Future studies will expand enrollment into larger multicenter cohorts, collect more cases with severe coronary calcification or inconclusive CCTA findings for adequately powered subgroup analyses, and further develop individualized diagnostic strategies. Additional work will also evaluate the real-world impact of the fusion model on clinical decision-making, patient outcomes, and healthcare resource utilization.

## 6. Conclusions

In conclusion, combining MCG with CCTA through ML significantly improves the detection of functionally significant myocardial ischemia compared to either modality alone. By leveraging the complementary strengths of electrophysiological assessment and anatomical imaging, this approach enhances diagnostic accuracy and achieves a better balance between sensitivity and specificity. Clinically, it offers a promising noninvasive tool for ischemia evaluation, with the potential to refine risk stratification and reduce reliance on invasive procedures.

## Data Availability

The datasets used during the current study are available from the corresponding author on reasonable request.

## Abbreviations

CCTA, coronary computed tomography angiography; MCG, magnetocardiography; CAD, coronary artery disease; FFR, fractional flow reserve; CT-FFR, CT-derived FFR; ICA, invasive coronary angiography; CFD, computational fluid dynamics; ML, machine learning; ECG, electrocardiography; SQUID, superconducting quantum interference devices; SERF, spin-exchange relaxation-free; CACS, calcium score; BMI, body mass index; TC, total cholesterol; TG, triglyceride; HDL-C, high-density lipoprotein cholesterol; LDL-C, low-density lipoprotein cholesterol; PIV, pan-immune-inflammation value; LVIDd, left ventricular internal dimension in diastole; LVIDs, left ventricular internal dimension in systole; FS, fractional shortening; LVEF, left ventricular ejection fraction; LR, logistic regression; SVM, support vector machine; RF, random forest; NB, naive bayes; XGBoost, extreme gradient boosting; PPV, positive predictive value; NPV, negative predictive value; IQR, interquartile range; ROC, receiver operating characteristic; LAD, left anterior descending artery; LCX, left circumflex artery; RCA, right coronary artery; CI, confidence interval; AUC, area under the receiver operating characteristic curve; SHAP, Shapley additive explanations.
