# Supplementary material for: Integration of Magnetocardiography and Coronary Computed Tomography Angiography With Machine Learning for Detection of Functionally Significant Myocardial Ischemia
Source: Rev Cardiovasc Med. 2026 Jun 8;27(6):47473. doi: 10.31083/RCM47473 (PMC13339176; doi:10.31083/RCM47473)
Supplement: Supplementary file 1 [file 2153-8174-27-6-47473-s1.zip › Supplementary Material.docx]

**Supplementary Table 1. Extracted MCG parameters.**

| Classification | Parameter | Description |
| --- | --- | --- |
| Basic electrophysiological parameters | PR interval | Interval from onset of P wave to onset of QRS complex |
|  | QRS interval | Duration of the QRS complex |
|  | QT interval | Interval from onset of QRS complex to end of T wave |
|  | QTc interval | Heart-rate–corrected QT interval |
|  | ST segment | ST-segment level/deviation relative to isoelectric baseline |
| Magnetic field strength and vector parameters | R/T magnetic strength ratio | Ratio of R-wave to T-wave magnetic field strength |
|  | T-wave peak positive/negative magnetic strength ratio | Ratio of positive to negative magnetic field strength at T peak |
|  | MCV amplitude (at R-wave peak) | Amplitude of magnetic current vector at R-wave peak |
|  | TCV angle (at R-wave peak) | Angle of total current vector at R-wave peak |
|  | MCV amplitude (at T-wave peak) | Amplitude of magnetic current vector at T-wave peak |
|  | TCV angle (at T-wave peak) | Angle of total current vector at T-wave peak |
| Spatial vector metrics | Dipole distance (at R-wave peak) | Distance between positive and negative dipole centers at R peak |
|  | Dipole distance (at T-wave peak) | Distance between positive and negative dipole centers at T peak |
|  | Dipole angle (at R-wave peak) | Orientation angle of the dipole at R-wave peak |
|  | Dipole angle (at T-wave peak) | Orientation angle of the dipole at T-wave peak |
|  | Half T-wave vector distance | Distance of the vector at half-T-wave time point |
|  | Half T-wave vector angle | Orientation angle of the vector at half-T-wave time point |
|  | Angular change of vector from half T-wave to T-wave peak | Change in vector angle from half-T-wave to T-wave peak |
| Composite parameters and risk assessments | Composite MCG parameter | Composite index derived from multiple MCG parameters |
|  | Composite MCG vector | Composite vector-based index derived from MCG metrics |
|  | MCG grade | Ordinal grading of ischemic burden based on MCG findings |
|  | Risk of persistent atrial fibrillation | Estimated risk score for persistent atrial fibrillation |
|  | Conduction block | Presence of MCG pattern suggesting atrioventricular block |

Supplementary Table 2. Extracted CCTA parameters.

| Classification | Parameter | Description |
| --- | --- | --- |
| Coronary plaque assessment | Plaque composition (vessel-level) | Vessel-level plaque composition recorded for LAD, LCX, and RCA as no plaque, non-calcified, calcified, or mixed |
| Coronary calcification | Coronary artery calcium score (CACS) | Agatston calcium score |
| Perivascular fat metric | Fat attenuation index (FAI) | Perivascular fat attenuation index measured for LAD/LCX/RCA in systolic and diastolic phases |
| Contrast attenuation metric | Transluminal attenuation gradient (TAG) | Transluminal attenuation gradient measured along LAD/LCX/RCA in systolic and diastolic phases |
| Cardiac chamber parameter | Left atrial volume | Left atrial volume measured in systolic and diastolic phases |

**Supplementary Table 3. Performance of all machine learning models.**

| ML models | AUC(95% CI) | Accuracy | Specificity | Sensitivity | PPV | NPV | F1-scrore |
| --- | --- | --- | --- | --- | --- | --- | --- |
| MCG Model |  |  |  |  |  |  |  |
| LR | 0.682 (0.615-0.749) | 0.636 | 0.678 | 0.561 | 0.491 | 0.736 | 0.524 |
| RF | 0.754 (0.691-0.816) | 0.745 | 0.853 | 0.551 | 0.675 | 0.774 | 0.607 |
| NB | 0.722 (0.656-0.787) | 0.720 | 0.808 | 0.561 | 0.618 | 0.769 | 0.588 |
| SVM | 0.699 (0.630-0.768) | 0.695 | 0.751 | 0.592 | 0.569 | 0.769 | 0.580 |
| XGBoost | 0.769 (0.708-0.829) | 0.731 | 0.797 | 0.612 | 0.625 | 0.788 | 0.619 |
| CCTA Model |  |  |  |  |  |  |  |
| LR | 0.686 (0.613-0.758) | 0.727 | 0.808 | 0.582 | 0.626 | 0.777 | 0.603 |
| RF | 0.725 (0.661-0.790) | 0.709 | 0.785 | 0.571 | 0.596 | 0.768 | 0.583 |
| NB | 0.743 (0.677-0.808) | 0.742 | 0.814 | 0.612 | 0.645 | 0.791 | 0.628 |
| SVM | 0.708 (0.642-0.774) | 0.658 | 0.672 | 0.633 | 0.517 | 0.768 | 0.569 |
| XGBoost | 0.755 (0.692-0.818) | 0.731 | 0.785 | 0.633 | 0.620 | 0.794 | 0.626 |
| Combine Model |  |  |  |  |  |  |  |
| LR | 0.781 (0.724-0.838) | 0.738 | 0.785 | 0.653 | 0.627 | 0.803 | 0.640 |
| RF | 0.814 (0.758-0.869) | 0.778 | 0.819 | 0.704 | 0.683 | 0.833 | 0.693 |
| NB | 0.816 (0.763-0.869) | 0.760 | 0.842 | 0.612 | 0.682 | 0.797 | 0.645 |
| SVM | 0.771 (0.711-0.831) | 0.738 | 0.808 | 0.612 | 0.638 | 0.790 | 0.625 |
| XGBoost | 0.829 (0.773-0.885) | 0.800 | 0.853 | 0.704 | 0.726 | 0.839 | 0.715 |

LR, Logistic Regression; RF, Random Forest; NB, Naive Bayes; SVM, Support Vetor Machine; XGBoost, Extreme Gradient Boosting.

Supplementary Table 4. Subgroup performance of the combined MCG-CCTA XGBoost model.

| Subgroup | Level | n | AUC(95% CI) |
| --- | --- | --- | --- |
| Overall |  | 275 | 0.829 (0.773–0.885) |
| Hypertension | No | 100 | 0.832 (0.734–0.916) |
|  | Yes | 175 | 0.743 (0.662–0.818) |
| History of smoking | No | 228 | 0.735 (0.661–0.805) |
|  | Yes | 47 | 0.864 (0.745–0.960) |
| Sex | Female | 114 | 0.731 (0.602–0.853) |
|  | Male | 161 | 0.767 (0.695–0.844) |
